# Sex hormone influence on female-biased autoimmune diseases hints at puberty as an important factor in pathogenesis

**DOI:** 10.3389/fped.2023.1051624

**Published:** 2023-01-30

**Authors:** Qianfan Yang, Kameron Kennicott, Runqi Zhu, Jooyong Kim, Hunter Wakefield, Katelyn Studener, Yun Liang

**Affiliations:** ^1^ School of Medicine and Public Health, Medical Microbiology and Immunology, University of Wisconsin-Madison, Madison, WI, United; ^2^ Departments of Physiology and Pharmacology and Toxicology, Michigan State University, East Lansing, MI, United States

**Keywords:** puberty, autoimmune diseases, sex hormones, immunity, immunometabolism

## Abstract

The majority of autoimmune diseases affect more women than men, suggesting an important role for sex hormones in regulating immune response. Current research supports this idea, highlighting the importance of sex hormones in both immune and metabolic regulation. Puberty is characterized by drastic changes in sex hormone levels and metabolism. These pubertal changes may be what forms the gulf between men and women in sex bias towards autoimmunity. In this review, a current perspective on pubertal immunometabolic changes and their impact on the pathogenesis of a select group of autoimmune diseases is presented. SLE, RA, JIA, SS, and ATD were focused on in this review for their notable sex bias and prevalence. Due to both the scarcity of pubertal autoimmune data and the differences in mechanism or age-of-onset in juvenile analogues often beginning prior to pubertal changes, data on the connection between the specific adult autoimmune diseases and puberty often relies on sex hormone influence in pathogenesis and established sex differences in immunity that begin during puberty.

## 1. Introduction

The regulatory effect of sex hormones on human immunity is a major focus in the field of immunology due to the differences men and women experience in the susceptibility and prognosis of various immune disorders. Emerging results from immunometabolic research show that metabolic dysregulation also influences immune disorders, especially autoimmunity. In this paper, both sex hormone and metabolic influence on autoimmunity will be discussed, with their pubertal changes in context. SLE, RA, JIA, SS, and ATD were chosen to explore these concepts because of their sex bias towards women and high prevalence.

To understand the effects of sex hormones on the immune system during adolescence for both sexes, the fluctuation of estrogen, progesterone, and testosterone and their physiological effect on metabolism will be reviewed. Next, the effects of puberty in shaping one's immune system by regulating immune cell diversity and cytokine production will be summarized. Furthermore, a summary of immunoendocrine and immunometabolism changes with regard to sex hormones during puberty and a novel perspective in understanding autoimmune diseases will be made.

A comprehensive traditional literature search was conducted for original studies using terms such as puberty, adolescence, juvenile, sex hormones, immunity, cytokines, autoimmunity, autoantibodies, immunometabolism, systemic lupus erythematous, rheumatoid arthritis, Sjögren's syndrome, juvenile lupus, juvenile idiopathic arthritis, autoimmune thyroid disease, Grave's disease, obesity, and diabetes. Searches for cell types involved in immunity were done individually (e.g., T cell, B cell, macrophage) as well as collectively by specific functions in immunity (e.g., breaking tolerance, signaling, T_h_1/T_h_2 response, APC). Search terms were either used individually or in some combinatorial permutation with each other. Additionally, search of cited sources within found studies was performed.

## 2. Hormone and metabolite changes in puberty

Puberty is an inevitable and irreducible process that every adolescent will go through before becoming an adult. Pubertal changes include, but are not limited to, changes in physical appearances, insulin sensitivity, emotional information processing, and hormone levels (1–3). While there are many pubertal changes, the changes in hormone levels emphasize sex differences represented by the pathogenesis of autoimmune diseases.

In addition to driving physical changes in sex organs, sex hormones are known for a wide range of functions, including controlling one's diet, emotional processing, and regulating immune responses in the body (4). Testosterone, a male sex hormone that increases in boys during puberty, was found to affect cognitive sensitivity of adolescents (5). Boys in the early pubertal stage with greater testosterone levels chose immediate rewards when compared to boys with lower testosterone levels, who were more likely to choose greater long-term rewards (5). Estradiol, a primary form of estrogen, showed anti-depressive properties by increasing reactions of anxiety and depression in estradiol knockout mice compared to their wild-type counterparts (6, 7). Consistent with their key roles in driving pubertal changes, the levels of testosterone, estrogen, and progesterone change in adolescent boys and girls during puberty. The average testosterone level of boys increased 250-fold between 6 and 20 years old while girls presented a 15-fold increase (8). Meanwhile, the average estradiol level in adolescent girls showed a 15-fold increase between 7 and 23 years old compared to 3-fold increase in boys between 7 and 18 years of age (9). Intriguingly, studies have revealed a critical role of progesterone in the male reproductive system (10, 11). Specifically, progesterone enhances sperm movement by activating the CatSper channel, a mobility by influx of Ca^2+^ (12, 13). With progesterone classically defined as a female hormone, its function in male reproductive events supports the idea that puberty is a complex event that cannot be defined solely by a single sex hormone.

Besides sex hormones, metabolic hormone levels can also exhibit sex-specific changes during puberty. Leptin, a hormone that is responsible for balancing food intake, showed sex-biased changes of its level during puberty. While leptin is associated with energy use and body fat distribution, rapidly increasing in pre-pubertal stage and decreasing to its basal level after puberty, female leptin levels remained higher than males after puberty (14). In particular, a study that examined blood samples of healthy adult men and women found that women had 2–3 times greater circulating leptin levels, consistent with the observation that women generally have a greater percentage of body fat than men (15, 16). Similarly, adiponectin, a hormone known to be a potential biomarker for metabolic syndromes, also displayed sex differences during puberty. Studies demonstrated that levels of adiponectin in boys decreased as puberty progressed whereas almost no change in adiponectin levels was observed in girls (17, 18). Growth hormone is also vital in physical development during puberty, but sex-related differences are yet to be described in detail (19, 20). Although we found that various hormone levels change during puberty, there was not enough data to support links to sex-biased autoimmune prevalence from leptin, adiponectin, and growth hormones. Because studies on hormonal regulation in autoimmunity mostly focus on sex hormones rather than puberty, more studies relating these hormones to the immune system and autoimmunity would be required to draw conclusions.

Hormonal changes can also induce metabolic disparity throughout the body, resulting in metabolic imbalance that may influence autoimmunity. An example of this can be seen with how testosterone affects energy metabolism (21–23). While testosterone is associated with weight gain and energy metabolism, male rats who had greater levels of testosterone than their female counterparts demonstrated greater food intake and weight gain at pre-pubertal stage along with reaching obesity earlier in development (10). Other than obesity, testosterone also displayed that it promotes protein synthesis and rapid muscle development in boys during puberty (21, 24). Many diseases involve dysregulation of protein metabolism. Cytokines and C-reactive protein (CRP) are notable immune proteins that, when dysregulated, are core components in the pathogenesis of autoimmune diseases. Therefore, hormonal changes during puberty may affect metabolism, and through its dysregulation promote autoimmune pathogenesis.

Puberty is a time dominated by hormonal changes. These changes, as discussed, have a broader impact than changes in outward appearance. Sex hormones in particular are implicated not only in sexual dimorphism, but in metabolic disorders and autoimmunity as well. It stands to reason that puberty, the time of greatest change in sex hormone levels, may be the start of their influence towards these diseases. To see if this correlates with data on pubertal immune systems, we also evaluated pubertal changes in the various elements of immunity.

## 3. Immune system functionality during puberty

Sexual dimorphism becomes more visibly apparent during puberty. Boys' voices deepen and testes enlarge, while girls begin to grow breasts and start menstruation. Underneath these visible changes are perhaps more pertinent ‘invisible’ differences in the immune system. Here the components of immunity will be compared between sexes: immune cell abundance and function, as well as cytokine and chemokine signaling.

Longitudinal studies that include profiling immune cell abundance and function between sexes during puberty are few and usually do not focus on sexual disparities or puberty. From what has been gathered, there are some comparisons of note (Table 1). Starting with innate immunity, one such difference is in monocytes in the peripheral blood. Adolescent girls have significantly less monocytes than adolescent boys but show similar maximal cytotoxic capacity when stimulated, and the significant sexual disparity is lost upon adulthood (28, 29). A small study from 1985 states that natural killer (NK) cell cytotoxicity decreases from childhood through adolescence to adulthood for females while males experience the opposite (30). Meanwhile, relative abundance data shows that there is no significant difference in NK cells between sexes in adolescence (28, 29). A study using a rat model reinforces both studies, noting the increase in female lung tumor metastases as a result of decrease in the antitumor function of NK cells (30). While these studies, like most, focused on the antitumor and antiviral capacities of NK cells, the immunoregulatory effects of NK cells like NK-DC crosstalk and antigen presentation have yet to be described in detail during puberty. In contrast to the animal model support of NK cell data, granulocytes show no significant differences in abundance in girls and boys (28, 31), but do appear to have sexually dimorphic abundance and function in mouse models. Male mice showed greater differentiation and release of myeloid cells from bone marrow coupled with an increase in inhibition of granulocyte apoptosis vs. their female counterparts in a sepsis model (32). Whether this incongruity is due to species differences, sample localization, or peripheral blood vs. bone marrow respectively, is unclear and worthy of consideration in future studies.

**Table 1:** An overview of immune system changes during puberty.

Cell Type	Abundance changes and functionality during lifespan
Monocytes	Less in females than males, but similar cytotoxic capacity. Abundance disparity lost upon adulthood (28, 29).
Natural killer cells	No difference in abundance between males and females. Cytotoxicity decreases in females (28–30).
Macrophages	Macrophages help develop breasts during puberty. In males, interstitial and peritubular testicular macrophages safeguard male fertility (34–36).
Plasmacytoid dendritic cells (pDCs)	Female pDCs produce more IFN*α* than males when stimulated. Initially thought to be due to estrogen, it is actually primarily driven by × chromosome number (37–39).
T cells	Suggested differences in CD4:CD8 and T_h_1/T_h_2 ratios, but no statistically significant data to support any T cell differences (28, 39).
B cells	Males show increases in B cells during puberty, but this difference equalizes in adulthood and reverses in old age. There are hints towards heightened male tolerance training with increased T-cell receptors as well, while females show higher titers of autoreactive antibodies which suggest the opposite (28, 31, 40–45).

While the aforementioned innate immune cells show potential differences in function between the sexes during puberty, macrophages have been identified as key players in the development of sex-specific organs like mammary ducts (33). Estrogen upregulation of CCR1 on macrophages makes them more responsive to CCL7, which is modulated by ACKR2 on stromal fibroblasts, all to attract trophic macrophages to the breast and develop the ductal epithelium at terminal end buds (34, 35). Likewise, in males, a special role exists for macrophages in the testes. Interstitial and peritubular testicular macrophages (tM*Φ*) act to safeguard male fertility by immunosuppression through preferential expression of IL-10 and antigen processing genes, respectively (36). Whether or not these sex-specific roles for macrophages during pubertal development influence the female bias towards autoimmunity requires study, but it does highlight how sex differences play pivotal roles in the immune system and puberty.

Plasmacytoid dendritic cells (pDCs) from females have been shown to produce more IFN*α*, an inflammatory cytokine, than males when stimulated. This difference was determined to be estrogen receptor (Esr1) dependent (37) and therefore potentially linked with puberty; but by controlling estradiol concentration and utilizing data from transgender and Turner's syndrome individuals, a recent study showed that it may be primarily driven by X-chromosome number (38). Although immunity seems to be highly tied to sex hormones, this instance shows that there is nuance in the mechanisms underpinning what exactly drives the connection and how impactful changes in sex hormones may or may not be. Since X-chromosome numbers do not change during the lifespan, the impact on IFN*α* levels during puberty may not be pertinent to pubertal development on autoimmunity nor be as strongly regulated by estrogen as previously suggested. Thus, the nuances of each modulation in immune cell function need to be explored in depth before the driving factors can be assigned.

Adaptive immunity also shows sex-biased trends that may be impacted by pubertal development, though that impact is highly dependent on cell type. Initial studies with T-cell subset data showed an increasing difference in male and female CD4:CD8 ratio, as well as girls starting to have more Th1 cells and less Th2 cells at the end of puberty (ages 17–19) and onward into adulthood (39). These data, however, were unable to reach statistical significance (*p* = 0.11) and more recent competing data asserts that this disparity is less pronounced *via* higher relative CD8 levels and also equalizes upon adulthood (28). Continuing with the theme, B-cells show a larger increase in males during puberty relative to the total percentage of leukocytes (28, 31), though this difference once again appears to equalize into adulthood and even reverse in old age (40). Interestingly, the increase in the mouse equivalent of MHC class II TCR is coupled with the relative increase in B-cells in adolescent males and might suggest an increase in immune tolerance training. A comparison of tolerogenic B-cell abundance during adolescence and hormonal influence over the negative selection of autoreactive B-cells (41, 42) to determine which has more impact on sex-biased tolerance and autoimmunity may explain this phenomenon. A potential result of this may be found in antinuclear antibodies (ANA), which increase in prevalence and titer during puberty, especially in females (43). Similarly, autoreactive anti-thyroid antibodies TGAb and TPOAb spike during the first year of puberty in females and are associated with thyroid nodules and Hashimoto's disease (44, 45).

Cytokines are the signals that modulate immunity. They can either be proinflammatory or anti-inflammatory, though under certain circumstances a proinflammatory cytokine may exhibit anti-inflammatory function and vice versa. When dysregulated, proinflammatory cytokines contribute to autoimmunity largely through inflammation and overactivation of innate immune cells. In contrast, dysregulation of anti-inflammatory cytokines may disrupt tolerance or the negative feedback mechanisms that prevent inflammation and immune cell activation (46). Studying the effects of cytokines in the pathogenesis of autoimmune diseases is difficult because many cytokines are pleiotropic and redundant, so identifying the role of a particular cytokine in a particular disease requires controlling for lots of variables. Perhaps as a result of this, *in vivo* human data on cytokine levels during puberty is sparse. To fully test the effect of a cytokine generally requires additional *in vitro* models that have been engineered to match localized immune cells or tissue, or it requires animal testing. Both of which have their own drawbacks and collectively require a lot of time and money to fit the scope of understanding the full extent of cytokine function. Beyond that, simulating puberty is usually reduced to treatments with sex hormones. The nuances of other more subtle changes during puberty may be missed if not also considering the role of growth hormones or other metabolites *in vitro*.

Some major proinflammatory cytokines include IL-1, TNF*α*, IL-6, IFN*γ*, IL-12, IL-18 (CXCL8), G-CSF and GM-CSF (47). These work by activating local tissue inflammatory responses through cytokine-mediated amplification of chemokine release, endothelial cell adhesion molecule upregulation, slowing blood flow, and increasing vascular permeability (48). IL-1, TNF*α*, and IL-6 specifically are noted as potent mediators of inflammation. IL-1 has been shown to confer advantage against sepsis and dissemination of infection, and when challenged in female vs. ovariectomized mice, it showed association with estrogen. Further testing revealed that this was due to an increase in IL-1 production by estrogen stimulation on peripheral blood macrophages (49). Likewise, TNF*α* and IL-6 both show repression *via* the unbound estrogen receptor and are therefore induced with estrogen stimulation (50). Meanwhile, testosterone has been shown to attenuate TNF*α* release in rats (51). However, in typical pubertal procession, proinflammatory cytokine TNF*α* and chemokine IL-8 serum levels decrease throughout adolescence, regardless of sex (52). This could be a result of tight regulation, or it may mean that increasing estrogen and testosterone levels are insufficient to describe the role of TNF*α* in sex differences during pubertal immunity. In the case of IFN*γ*, estrogen's positive regulation of IFN*γ* correlates with sex differences during puberty, with adolescent girls starting to show significantly more by age twelve (53, 54).

Major anti-inflammatory cytokines include IL-1 Ra, IL-4, IL-10, IL-11, and IL-13. IL-10 is noted as potent, due to its ability to downregulate proinflammatory cytokine expression of IL-1, TNF*α*, IL-6 and other proinflammatory cytokine receptors as well as upregulate endogenous anti-cytokines (55). Although there is no pubertal data comparing sexes for any of these cytokines, IL-10 has been positively associated with increased estradiol, leading to increased antibody production (56). IL-10 expression also increases in mouse CD4 T cells when stimulated with testosterone (57). As both testosterone and estrogen upregulate IL-10 in some capacity, it is unlikely that IL-10 drives the sex bias of autoimmunity. Data comparing the efficacy of these effects with adolescent levels of cytokines would be more conclusive to that end. Mouse models show more stark differences in the regulation of IL-11 and IL-4. IL-11 shows upregulation by estrogen and downregulation by progesterone, whereas estrogen shows no significant effect on IL-4 expression in mouse splenocytes, yet testosterone upregulates it (57–59). IL-13 is noted for its role in inhibiting estrogen-induced breast cancer (60).

Overall, sex hormones have a large effect on the regulation of cytokine production. While estrogen may often provide females a stronger immune response by way of upregulated cytokine expression, it is not only in favor of the proinflammatory ones. Even in the cases where there is no clear difference between sexes of normal cytokine levels during puberty, there are implications that different regulatory mechanisms are maintaining that balance between sexes due to estrogen's influence. This may be the case of TNF*α*, which is implicated in many autoimmune disorders. Maintaining tight regulation of TNF*α* may require more inhibitors like IL-10 for maturing women and be part of their susceptibility to autoimmunity.

Testosterone, estrogen, and progesterone have been proven to be important sex hormones that play a vital role in developing sex-specific characteristics and directly or indirectly regulating immune pathways. Similarly, hormones such as leptin and adiponectin, which regulate metabolic functions, also show sex difference during puberty. This sex-specific role of hormones, along with their immunomodulatory role with cytokines, lends itself to the idea that hormone shifts, metabolic changes, and immune system changes during puberty are important to autoimmune pathogenesis. Cytokines are important regulators of immune cell function, and sex hormones have been shown to influence their production, efficacy, and function. Both hormone abundance fluctuation and immune cells abundance fluctuation during puberty may induce the variance in cytokines and subsequently may contribute to autoimmunity. These results hypothesize that metabolic changes and hormone shifts during puberty may make someone more prone to develop autoimmune disease post puberty. In the next parts of this review article, specific autoimmune responses and diseases will be discussed in this context.

## 4. Pathogenesis of autoimmune diseases

### 4.1. Sex hormones and autoimmune diseases

The immunomodulatory effect of sex hormones suggests a link between puberty, sex bias in autoimmune diseases, and their pathogenesis. Here, we will review established roles of sex hormones in Systemic lupus erythematosus (SLE), rheumatoid arthritis (RA), Sjögren's Syndrome (SS), and autoimmune thyroid disease (ATD) all of which exhibit increased prevalence in females. Since most autoimmune diseases present after puberty, pubertal autoimmune data is sparse. Therefore, investigating the role of puberty in these diseases relies heavily on sex hormones, which both change greatly during puberty and modulate immunity, and on sexual dimorphism between immune systems that begins during puberty. SLE, RA, SS and ATD are prime examples for evaluating the relationship between puberty and autoimmunity because of the role sex hormones have on their pathogenesis. Extrapolating relationships from sex hormones to puberty is not sufficient for truly developing our understanding of the pathogenesis of these diseases, but it may invigorate research into a pubertal basis for the beginnings of autoimmune pathogenesis as well as its sex bias.

#### 4.1.1. Systemic lupus erythematosus (SLE)

SLE is a chronic autoimmune disease that can present as widespread inflammation in the joints, skin, brain, lungs, kidneys, and blood vessels. Acute “flares” of inflammation are also possible, and both chronic and acute inflammation can lead to tissue damage. It is widely believed that environmental, hormonal, and genetic factors can contribute to disease onset and severity. However, the higher prevalence of SLE in females draws attention to the relationship between sex hormones and the immune system. Due to limited pubertal autoimmune data, sex hormone influence on autoimmunity during adulthood often must be used to infer a relationship arising from sex hormone changes during puberty.

17*β*–estradiol, the E2 form of estrogen, has been suggested as an explanation for dimorphism in autoimmune diseases including SLE. While 17*β*–estradiol and prolactin act as enhancers of humoral immunity, testosterone, and progesterone act as suppressors (64, 65). 17*β*–estradiol increases cell growth and proliferation marker expression by activating the NF*κ*B pathway, which is also an important inflammatory mediator (66). Indeed, SLE patients have higher amounts of pro-inflammatory cytokines IL-6 and IL-17 with plasma estradiol levels being positively correlated with IL-6 in SLE patients. In addition, IL-6 and estradiol changes are associated with higher SLEDAI scores. Estradiol addition to healthy control peripheral blood mononuclear cells does not dramatically influence the level of IL-6, however, the fluctuation of IL-6 levels in SLE patients are dependent on estradiol stimulation. The relationship between estradiol and IL-6 is interesting, especially when we consider the transformation in its expression during puberty. A longitudinal study conducted by researchers in Brazil observed the cytokine profile of juvenile SLE patients, where 92-percent of the participants were female with an average age of 15. In their findings, they took note of a significant increase in blood levels of IL-6 and IL-10 compared to healthy counterparts. Likewise, IL-6, IL-10, and IL-17a had greater expression in juvenile patients with active SLE states unlike patients with inactive disease (117). For both childhood SLE and adult, it would appear that IL-6 promotes inflammation, while IL-10 promotes autoantibody production. The increase in estrogen during puberty and the previously described relationship between estrogen and these cytokines suggest a link between adolescence and disease.

In addition, IFN*γ*, IL-18, IL-8, SCF and IL-23 secretions are higher in SLE patients compared to healthy control by the induction of 50 pg/ml of estradiol (62). While plasma estradiol levels have no obvious difference between SLE and healthy patient populations, estrogenic metabolites, such as 2-hydroxyestrogen and 16-hydroxyestrone and their derivatives, have been found to differ in levels amongst healthy and SLE patient populations (63). In a macaque study, there is fluctuation in the number of circulating, immunoglobulin-secreting B cells during the menstrual cycle when the amount of estrogen and progesterone changes, which implicates the modulation of autoantibody secretion by sex hormones (67–70).

Estradiol stimulation correlates with IL-6 level in SLE patients; moreover, the concentration of IL-6 has positively impacted SLE's severity. As seen with macaques, fluctuation in secreted B-cells is also linked to estrogen and progesterone increases (70). Because these hormones drastically increase during puberty for females, their effect on proinflammatory cytokines and autoantibody-producing B-cells for SLE patients may indicate puberty as a beginning in SLE pathogenesis.

Progesterone is a major sex hormone during menstruation and pregnancy. Like estradiol, progesterone also functions in maturation during puberty (71). However, progesterone opposes estradiol in its immunomodulatory effects. Estradiol is, in many cases, viewed as an enhancer of immune processes while progesterone is considered immunosuppressive and may have the ability to neutralize the effect of estrogens (72). For example, while estradiol may enhance SLE severity by modulating T helper cell cytokine secretion, progesterone has been shown to ease pro inflammatory signaling by inhibition of Th1 and Th17 cells (73).

Further investigation of estradiol and progesterone in immunity can give insight into their roles in autoimmune disease development.

#### 4.1.2. Rheumatoid arthritis (RA) and juvenile idiopathic arthritis (JIA)

Adult onset RA and JIA are similar yet distinct autoimmune diseases that show connections to sex hormones and elucidate an important change during puberty that may lead to the sex bias in autoimmune prevalence. Both diseases present with persistent chronic inflammation of the joints, synovial invasion by lymphocytes, plasma cells, and macrophages, and hyperproliferation of both synoviocytes and lymphocytes (104). Many lymphocytes in the synovial fluid are clonally expanding activated T-cells, with a mixed Th1/Th2 response (115, 116). Characteristically of autoimmune diseases, both JIA and RA rely on the breakdown of immunogenic tolerance. The common risk factor for many autoimmune diseases, human leukocyte antigen (HLA) is the primary suspect for this role. Polymorphisms in HLA, such as HLA-DRB1*04 alleles in RA and HLA-DR4 in JIA, are strong predictive markers for rheumatoid factor seropositivity and increased disease susceptibility and severity (107). Likewise, analysis of TCR's in the synovial fluid suggest that recognition of a limited group of autoantigens drive T-cell hyperproliferation, B-cell autoantibody production, and form the basis for the distinct subtypes in JIA and RA (109, 110). Though these diseases both appear to be autoantigen driven, another important factor to their pathogenesis is immunoregulation by hormones.

While the proinflammatory effect of estrogen is well researched in regard to female sex bias towards autoimmunity, the protective effects of androgens like testosterone and dehydroepiandrosterone (DHEA) are equally important. DHEA is the second largest in production among all androgens and its secretion is highly dependent with age (74). Peak production of DHEA happens in a woman's twenties and thirties before production begins to decline. DHEA is responsible for regulating a list of cytokines by suppressing Th1 and Th2 secretion function. This regulatory mechanism leads to a decreased release of proinflammatory cytokines such as IFN*γ* (75). Consequently, RA pathways that trigger autoreactive macrophage activation are linked to increased IFN*γ* production and would be negatively regulated by DHEA (77). In both male and female RA patients, DHEA levels are decreased, which may suggest that it plays a role in regulating disease onset and severity (76). JIA patients, on the other hand, show normal levels of DHEA compared to healthy children, but with closely related DHEA-S instead showing a 10-fold decrease in relative levels for both sexes (108).

DHEA concentration peaks at puberty and decreases post-puberty; it can be suggested that higher amounts of DHEA in puberty protect those genetically predisposed to RA by manipulating pro-inflammatory cytokine secretion. However, as females have much lower DHEA after puberty than males, the protection mechanism of DHEA is disappearing gradually in females and leads to the sex-bias in rheumatoid arthritis.

Testosterone is another powerful androgen that also declines with age (74). Lower androgen-to-estrogen ratios have been detected in both female and male RA patients compared to healthy controls (78, 79). Walecki et al. validated the effectiveness of testosterone treatment in elevating regulatory T cell (T_reg_ cells) population both *in vivo* and *in vitro*. T_reg_ cells are known for their ability in inhibiting proliferation and cytokine production in effector T cells. Because of T_reg_'s unique function in the immune system, they are believed to play an important role in preventing autoimmunity (80). In Yang's study, CD4 positive T cells are hyperproliferative in RA patients, leading to an increased amount of cytokine - producing effector cells (81). Linked back to the testosterone role stated in Walecki's study, it suggests that testosterone may help determining the pathogenesis of RA by regulating Treg and effector T cells which are responsible for cytokine production.

Androgens have been proven to greatly affect the immune system in both sexes (82). In males, androgens remain in a low activated state before puberty and significantly shift to high activated state when it highly impacts the system physiologically (83). Androgen level is usually low before puberty in girls (84). In general, men have significantly greater levels of androgen than women. The apparent difference in a protective androgen effect on RA between sexes could result from the increased gap in their androgen levels. While genetic risk factors and autoantigens appear to drive JIA and RA pathogenesis, androgens such as DHEA and testosterone appear to be a vital player in RA onset. The contrast between higher levels of androgen circulating in males post puberty vs. females may be an explanation for sex biases in RA and the other autoimmune diseases. Therefore, mechanisms by which these hormones decline from pubertal levels seem influential in autoimmune pathogenesis, especially RA.

#### 4.1.3. Sjögren's syndrome (SS)

Sjögren's syndrome is a chronic autoimmune disease with common symptoms of dry eyes and dry mouth. While the exact pathogenic mechanism of the disease is being elucidated, Sjögren's syndrome involve the dysfunction of salivary and lacrimal glands as well as increased levels of proinflammatory cytokines and auto-antibody levels (85, 86). Consistent with increased prevalence in middle-aged women, recent studies have suggested a key role of sex hormones in disease onset and progression.

In a study of ovariectomized mice, removal of the ovaries was found to increase the likelihood of SS like symptoms, such as apoptosis of epithelial cells. This symptom was improved after estrogen administration (87). In a study with 2,680 female Sjögren's syndrome patients, lower estrogen levels were associated with disease onset. It has been suggested that elevated estrogen levels prevent the development of Sjögren's syndrome (88). While salivary gland epithelial cells obtained from both Sjögren's patients and control groups expressed estrogen receptor alpha and estrogen receptor beta, patient cells showed reduced responsiveness to 17 beta-estradiol (86). Additionally, both dehydroepiandrosterone (DHEA) and dehydroepiandrosterone-sulfate (DHEA-S) show reduced levels in Sjögren's syndrome patients (89).

Testosterone can suppress some immune-related genes such as cathepsin S in lacrimal glands from female mouse models of Sjögren's syndrome. Cathepsin S is a lysosomal acidic protease highly expressed in lymphocytes and other MHCII - positive cells and is thought to play a role in the adaptive immune response (90, 91). The overexpression of Cathepsin S has been associated with various diseases, including arthritis, cancer, and cardiovascular disease. In Sjögren – prone mice, testosterone has been shown to lower the expression of cathepsin S and additional immune associated genes, resulting in decrease in disease severity (92, 93).

Post-pubertal males have significantly different hormone levels than females. Interestingly, males have much lower estrogen levels than females, however they don't have as high prevalence in Sjogren's syndrome. From a testosterone perspective, higher testosterone prevents expression of cathepsin S and protects males from Sjogren's syndrome. On the other hand, females are not equipped with higher testosterone to compensate for the disadvantage from estrogen. Combining estrogen and testosterone effects on the onset of Sjogren's syndrome, sex hormones greatly orchestrate the immune system functionality post-puberty. Since the sexual dimorphism in hormones arise from puberty, Sjogren's syndrome pathogenesis may also begin there.

#### 4.1.4. Autoimmune thyroid disease (ATD)

Autoimmune thyroid diseases (ATD)have been a leading reason for thyroid dysfunction. Grave's disease (GD) and Hashimoto's thyroiditis (HT) are two common presenting forms of ATD. Although clinical findings are distinct from GD (hyperthyroidism) and HT (hypothyroidism), they share a similar immune-mediated mechanism, which is characterized by the production of thyroid autoantibodies and thyroidal lymphocytic infiltration (94). Women have higher risk than men to develop ATD post-puberty, which might suggest that female pubertal changes play an important role in shaping the female immune system towards ATD (95).

Grave's disease is the most common type of autoimmune disease and is 10 times more prevalent in women (96, 97). Patients of GD have higher levels of thyroid hormone, suppressed thyroid - stimulating hormone (TSH), and detachable TSH receptor antibody (TSHRAb). It is well supported by these elevated laboratory results that GD is regulated by an antibody-mediated immune mechanism. It is a widely accepted idea that B cells are responsible for antibody production and play a pivotal role in pathogenesis of autoimmune disease (98). B cell activating factor (BAFF) as a pro-survival factor is responsible for regulating immune response by stimulating B cell maturation and differentiation (99). During autoimmunity, BAFF overexpression prevents autoreactive B cells from undergoing negative selection (98). It has been found that upregulated BAFF is linked to the onset of several autoimmune diseases, including GD. Cheng et al. confirmed that serum BAFF levels were greatly increased in GD groups compared to control groups in both sexes, suggesting serum BAFF protein is involved in thyroid function in GD patients. In addition, Cheng et al. found that exogenously introduced estradiol elevates BAFF protein level and thyroid function in male SAT mice. In summary, they concluded that estradiol is involved in the elevated BAFF level, therefore leading to pathogenesis of ATD (96).

Estradiol, an important form of estrogen, sees a significant change in its circulating amounts after puberty in males and females. Estradiol promotes BAFF protein transcription and dysregulated thyroid function in both male and females. Due to the possible interplay between estrogen and BAFF in GD development, it can provide a clue that pubertal sex hormone shifts in estrogen could be the driving factor for the higher incidence rate of autoimmune disease in females.

### 4.2. Immunometabolic and behavior modulation of autoimmunity

Recent studies suggest a connection between metabolic disorders and autoimmunity. These immunometabolic disorders are commonly regulated by sex hormones, indicating that puberty may also impact autoimmune pathogenesis indirectly through metabolism. Here, we discuss obesity and diabetes, as they are very common metabolic disorders that show hormone-mediation and an increased risk in autoimmunity.

#### 4.2.1. Obesity

The most common metabolic disorder, obesity, is tightly related with endocrine dysregulations in both sexes in childhood and adulthood. Obese boys present significantly lower levels of total testosterone and free testosterone than their non-obese peers (101). This could contribute to a decreased protective effect against autoimmunity. Inflammatory cytokines secreted by white adipose tissue, also known as adipokines, are considered the culprit of obesity-induced immune destruction (105, 111). The connection between obesity and autoimmunity is becoming progressively lucid. de Candia et al. have found that adipose inflammation fueled by abnormally accumulated adipose tissue and autoreactive T cells might be an early symptom for obese patients that develop autoimmune complications (106). This may be part of the reason that childhood obesity is a risk factor for RA, MS, type 1 diabetes (T1D), psoriatic arthritis, and Hashimoto thyroiditis [Obesity in autoimmune diseases: not a passive bystander]. While these discoveries show promising illustrations of the relationship between obesity and autoimmunity, the role of obesity, especially childhood obesity, on mechanisms of autoimmune pathogenesis requires further investigation.

#### 4.2.2. Diabetes

In addition to obesity, diabetes represents an established connection between metabolic and autoimmune disorders. Type I diabetes is known for autoreactive T cell-mediated destruction of pancreatic islet cells; meanwhile, type II diabetes (T2D) develop low grade inflammation that scars tissue by exacerbating other T2D pathologies, such as insulin resistance and atherosclerotic plaque development and destabilization, and has recently been considered an autoimmune disease by some studies (25, 26, 106).

Both T1D and T2D are implicated in the altering of TSH, leading to an increase in anti-thyroid autoantibodies and greater risks of ATD, especially so in T1D adolescents (61, 100). The exact mechanism by which diabetes influences TSH is unclear, but since metformin use is associated with greater risk of lower TSH in hypothyroid patients, decreased blood glucose or increased insulin response could be responsible for connecting diabetes and ATD (102). Since dysregulated sex hormones are implicated in the pathogenesis of both ATD and diabetes, and T1D adolescents show an increased risk in ATD, pubertal changes in sex hormones appear to affect autoimmunity both directly and indirectly.

With estrogen and testosterone being the two main sex hormones that regulate pubertal development, their correlation to diabetes onset and progression suggests that their drastic pubertal changes are important to diabetes pathogenesis (112). A lack of estrogen and testosterone is linked to increased risk of insulin resistance and earlier onset of diabetic disorders in both men and women (103, 113, 114). Results that show testosterone affects protein metabolism imply that it may also affect T2D by suppressing or enhancing immune genes related to inflammation, cytokine, and CRP levels, which are common indicators for autoimmune diseases (21, 27).

Since sex hormone levels change drastically with the onset of puberty, it is reasonable to suspect that changes within the body during adolescence hint at a greater impact on immunity later, and immunometabolic changes, such as obesity and diabetes, could either be avenues of increasing the risk of autoimmunity or accelerants towards its onset or severity. Therefore, prospective research should further inspect the role of adolescent sex hormone changes with relation to obesity, diabetes and other metabolic diseases with autoimmunity.

### 4.3. Puberty-Associated behavior change and autoimmunity

Other than hormonal changes, behavior shifts during adolescence and beyond are becoming a pronounced factor in determining disease expression and prognosis. For example, eating disorders are one of the most commonly troubling disorders in adolescents and young adults. In a large cohort study of over 900,000 individuals in Denmark, researchers confirmed previous findings that adolescents with eating disorders were at greater risk of developing autoimmune disorders. Conversely, it was observed that those with autoimmune disorders have greater risk for eating disorders (118, 119). While most research into eating disorders focuses on anorexia and bulimia, this study qualified individuals that met eating disorder standards but did not specify any disorder. These eating disorders were labeled as EDNOS and predicted a higher hazard of a subsequent autoimmune disease with gastrointestinal involvement, lending credence to the idea that an individual's gut microbiome and immunometabolism may be linked to puberty and autoimmunity. It is common knowledge that adolescents experience both emotional and behavioral dilemmas, resulting in higher stress and behavioral disorders which may in turn contribute to autoimmunity later in life. The exact mechanism by which stress, eating disorders, or other common adolescent behavioral changes may increase the risk of autoimmunity has yet to be explored, but the connection is there.

## 5. Conclusion

Many hormonal changes occur during puberty, especially with testosterone and estrogen, influencing sex-specific bodily changes. Alterations are also seen in the immune system, with notable changes in the abundance of T-cell subsets, monocytes and B-cells. Sex hormones have been shown to directly impact immune cell function, leading them to regulate many autoimmune diseases. Androgens are primarily protective against autoimmunity, while dysregulations in estrogens, too much or too little, appear to be linked with autoimmunity. In addition, these hormones further affect autoimmune pathogenesis through their regulation of metabolic pathways, seen in obesity and diabetes. The dramatic shift in the endocrine and immune systems during puberty may help explain the sexual dimorphism of autoimmune diseases and calls for greater scrutiny in how we address clinical management strategies in pre- and post-pubescent individuals.

## References

[1] Bunoti SN Tumwesigye NM Atuyambe L. Awareness of pubertal body changes among primary school children aged 10–14 years in eastern Uganda; challenges and opportunities. Reprod Health. (2022) 19:1. 10.1186/s12978-022-01466-y 35986331 PMC9392265

[2] Silk JS Siegle GJ Whalen DJ Ostapenko LJ Ladouceur CD Dahl RE. Pubertal changes in emotional information processing: pupillary, behavioral, and subjective evidence during emotional word identification. Dev Psychopathol. (2009) 21:1. 10.1017/s0954579409000029 19144220 PMC2629078

[3] Kelly LA Lane CJ Weigensberg MJ Toledo-Corral CM Goran MI. Pubertal changes of insulin sensitivity, acute insulin response, and *β*-cell function in overweight latino youth. J Pediatr. (2011) 158:3. 10.1016/j.jpeds.2010.08.046 20888012 PMC3039101

[4] Lühder F Lee D Gold R Stegbauer J Linker R. Small but powerful: short peptide hormones and their role in autoimmune inflammation. J Neuroimmunol. (2009) 217:1–2. 10.1016/j.jneuroim.2009.08.008 19748684

[5] Laube C Suleiman AB Johnson M Dahl RE Van den Bos W. Dissociable effects of age and testosterone on adolescent impatience. Psychoneuroendocrinology. (2017) 80:162–9. 10.1016/j.psyneuen.2017.03.012 28363134 PMC9068513

[6] Koss WA Einat H Schloesser RJ Manji HK Rubinow DR. Estrogen effects on the forced swim test differ in two outbred rat strains. Physiology; Behavior. (2012) 106:2. 10.1016/j.physbeh.2012.01.004 PMC3314120 22266677

[7] Dalla C Antoniou K Papadopoulou-Daifoti Z Balthazart J Bakker J. Oestrogen-deficient female aromatase knockout (ArKO) mice exhibit “depressive-like” symptomatology. Eur J Neurosci. (2004) 20:1. 10.1111/j.1460-9568.2004.03443.x 15245494

[8] Senefeld JW Lambelet Coleman D Johnson PW Carter RE Clayburn AJ Joyner MJ. Divergence in timing and magnitude of testosterone levels between male and female youths. JAMA. (2020) 324:1. 10.1001/jama.2020.5655 PMC7341166 32633795

[9] Bidlingmaier F Wagner-Barnack M Butenandt O Knorr D. Plasma estrogens in childhood and puberty under physiologic and pathologic conditions. Pediatr Res. (1973) 7:11. 10.1203/00006450-197311000-00006 4795966

[10] Shah C Modi D Sachdeva G Gadkar S Puri C. Coexistence of intracellular and membrane-bound progesterone receptors in human testis. J Clin Endocrinol; Metabolism. (2005) 90:1. 10.1210/jc.2004-0793 15509639

[11] Gadkar S Shah C Sachdeva G Samant U Puri C. Progesterone receptor as an indicator of sperm function. Biol Reprod. (2002) 67:4. 10.1095/biolreprod67.4.1327 12297552

[12] Strünker T Goodwin N Brenker C Kashikar ND Weyand I Seifert R The CatSper channel mediates progesterone-induced Ca2 + influx in human sperm. Nature. (2011) 471:7338. 10.1038/nature09769 21412338

[13] Lishko PV Botchkina IL Kirichok Y. Progesterone activates the principal Ca2 + channel of human sperm. Nature. (2011) 471:7338. 10.1038/nature09767 21412339

[14] Quinton ND Smith RF Clayton PE Gill MS Shalet S Justice SK Leptin binding activity changes with age: the link between leptin and Puberty1. J Clin Endocrinol; Metabolism. (1999) 84:7. 10.1210/jcem.84.7.5834 10404799

[15] Hellstrom L Wahrenberg H Hruska K Reynisdottir S Arner P. Mechanisms behind gender differences in circulating leptin levels. J Intern Med. (2000) 247:4. 10.1046/j.1365-2796.2000.00678.x 10792559

[16] Blaak E. Gender differences in fat metabolism. Curr Opin Clin Nutr Metab Care. (2001) 4:6. 10.1097/00075197-200111000-00006 11706283

[17] BöTtner A Kratzsch J Müller G Kapellen TM Blüher S Keller E Gender differences of adiponectin levels develop during the progression of puberty and are related to Serum androgen levels. J Clin Endocrinol; Metabolism. (2004) 89:8. 10.1210/jc.2004-0303 15292348

[18] Andersen KK Frystyk J Wolthers OD Heuck C Flyvbjerg A. Gender differences of oligomers and total adiponectin during puberty: a cross-sectional study of 859 danish school children. J Clin Endocrinol Metab. (2007) 92:5. 10.1210/jc.2006-2310 17284625

[19] Olney RC Prickett TCR Yandle TG Espiner EA Han JC Mauras N. Amino-Terminal propeptide of C-type natriuretic peptide and linear growth in children: effects of puberty. Testosterone, and Growth Hormone. J Clin Endocrinol & Metabolism. (2007) 92:11. 10.1210/jc.2007-0567 17684048

[20] Sotos JF Tokar NJ. Growth hormone significantly increases the adult height of children with idiopathic short stature: comparison of subgroups and benefit. Int J Pediatr Endocrinol. (2014) 2014:1. 10.1186/1687-9856-2014-15 PMC4114101 25075207

[21] Markofski MM Volpi E. Protein metabolism in women and men: similarities and disparities. Curr Opin Clin Nutr Metab Care. (2011) 14:1. 10.1097/mco.0b013e3283412343 21088570 PMC3176666

[22] Mutoh E Senba K Akieda-Asai S Miyashita A Poleni P Date Y. Sex differences in energy metabolism in pre-pubescent, early pubescent and adult rats. Obes Res Clin Pract. (2011) 5:2. 10.1016/j.orcp.2010.12.006 24331063

[23] Nunez AA Grundman M. Testosterone affects food intake and body weight of weanling male rats. Pharmacol Biochem Behav. (1982) 16:6. 10.1016/0091-3057(82)90048-x 7111353

[24] Tipton KD. Gender differences in protein metabolism. Curr Opin Clin Nutr Metab Care. (2001) 4:6. 10.1097/00075197-200111000-00005 11706282

[25] Procaccini C Carbone F Galgani M La Rocca C de Rosa V Cassano S Obesity and susceptibility to autoimmune diseases. Expert Rev Clin Immunol. (2011) 7:3. 10.1586/eci.11.18 21595595

[26] Mzimela NC Ngubane PS Khathi A. The changes in immune cell concentration during the progression of pre-diabetes to type 2 diabetes in a high-fat high-carbohydrate diet-induced pre-diabetic rat model. Autoimmunity. (2019) 52:1. 10.1080/08916934.2019.1575820 30776930

[27] Kolb H Mandrup-Poulsen T. An immune origin of type 2 diabetes? Diabetologia. (2005) 48:6. 10.1007/s00125-005-1764-15864529

[28] Valiathan R Ashman M Asthana D. Effects of aging on the immune system: infants to elderly. Scand J Immunol. (2016) 83:255–66. 10.1111/sji.12413 26808160

[29] Koopman MG Fleer A v.d. Schaaf D v.d. Meulen F Von Dem Borne K Engelfriet C. Male-female differences in the cytotoxic activity of human monocytes in vitro. Clin & Laborat Hematol. (1981) 3:45–50. 10.1111/j.1365-2257.1981.tb01308.x 7226721

[30] Horikoshi T. Significant differences in natural killer cell activity in children according to age and sex. Kurume Med J. (1985) 32:1. 10.2739/kurumemedj.32.63 3866876

[31] Bartlett JA Schleifer SJ Demetrikopoulos MK Delaney BR Shiflett SC Keller SE. Immune function in healthy adolescents. Clin Diagn Lab Immunol. (1998) 5:1. 10.1128/CDLI.5.1.105-113.1998 9455890 PMC121401

[32] Aoyama M Kotani J Usami M. Gender difference in granulocyte dynamics and apoptosis and the role of IL-18 during endotoxin-induced systemic inflammation. Shock. (2009) 32:4. 10.1097/SHK.0b013e31819c358a 19174744

[33] Coussens LM Pollard JW. Leukocytes in mammary development and cancer. Cold Spring Harb Perspect Biol. (2011) 3:3. 10.1101/cshperspect.a003285 PMC3039933 21123394

[34] Wilson GJ Fukuoka A Love SR Kim J Pingen M Hayes AJ Chemokine receptors coordinately regulate macrophage dynamics and mammary gland development. Development. (2020) 147:12. 10.1242/dev.187815 PMC7328164 32467242

[35] Pollard JW. Trophic macrophages in development and disease. Nat Rev Immunol. (2009) 9:4. 10.1038/nri2528 PMC3648866 19282852

[36] Mossadegh-Keller N Sieweke MH. Testicular macrophages: guardians of fertility. Cell Immunol. (2018) 330:120–5. 10.1016/j.cellimm.2018.03.009 29650243

[37] Griesbeck M Ziegler S Laffont S Smith N Chauveau L Tomezsko P Sex differences in plasmacytoid dendritic cell levels of IRF5 drive higher IFN*α* production in women. J Immunol. (2015) 195:11. 10.4049/jimmunol.1501684 PMC4654231 26519527

[38] Webb K Peckham H Radziszewska A Menon M Oliveri P Simpson F Sex and pubertal differences in the type 1 interferon pathway associate with both X chromosome number and Serum sex hormone concentration. Front Immunol. (2019) 9. 10.3389/fimmu.2018.03167 PMC6345344 30705679

[39] Tollerud DJ Ildstad ST Brown LM Clark JW Blattner WA Mann DL T-cell subsets in healthy teenagers: transition to the adult phenotype. Clin Immunol Immunopathol. (1990) 56:1. 10.1016/0090-1229(90)90172-M 2357861

[40] Márquez EJ Chung C Marches R Rossi R Nehar-belaid D Eroglu A Sexual-dimorphism in human immune system aging. Nat Commun. (2020) 11:751. 10.1038/s41467-020-14396-9 PMC7005316 32029736

[41] Tengstrand B Carlström K Felländer-Tsai L Hafström I. Abnormal levels of serum dehydroepiandrosterone, estrone, and estradiol in men with rheumatoid arthritis: high correlation between serum estradiol and current degree of inflammation. J Rheumatol. (2003) 30:11. 14677174

[42] Luckey D Medina K Taneja V. B cells as effectors and regulators of sex-biased arthritis. Autoimmunity. (2012) 45:5. 10.3109/08916934.2012.665528 PMC3720982 22429183

[43] Sperotto F Cuffaro G Brachi S Seguso M Zulian F. Prevalence of antinuclear antibodies in schoolchildren during puberty and possible relationship with musculoskeletal pain: a longitudinal study. J Rheumatol. (2014) 41:7. 10.3899/jrheum.130948 24737914

[44] Taubner K Schubert G Pulzer F Pfaeffle R Körner A Dietz A Serum concentrations of anti-thyroid peroxidase and anti-thyroglobulin antibodies in children and adolescents without apparent thyroid disorders. Clin Biochem. (2014) 47:1–2. 10.1016/j.clinbiochem.2013.09.017 24103918

[45] Xu W Huo L Chen Z Huang Y Jin X Deng J The relationship of TPOAb and TGAb with risk of thyroid nodules: a large epidemiological study. Int J Environ Res Public Health. (2017) 14:7. 10.3390/ijerph14070723 PMC5551161 28678169

[46] Elenkov IJ Chrousos GP. Stress hormones, proinflammatory and anti-inflammatory cytokines, and autoimmunity. Ann N Y Acad Sci. (2002) 966:290–303. 10.1111/j.1749-6632.2002.tb04229.x 12114286

[47] Cavaillon JM. Pro- versus anti-inflammatory cytokines: myth or reality. Cell Mol Biol. (2001) 47:4. 11502077

[48] Holdsworth SR Gan PY. Cytokines: names and numbers you should care about. Clin J Am Soc Nephrol. (2015) 10:12. 10.2215/CJN.07590714 25941193 PMC4670773

[49] Youssef H Stashenko P. Interleukin-1 and estrogen protect against disseminating dentoalveolar infections. Int J Oral Sci. (2017) 9:16–23. 10.1038/ijos.2016.61 28358036 PMC5379163

[50] An J Ribeiro RCJ Webb P Gustafsson J Kushner PJ Baxter JD Estradiol repression of tumor necrosis factor-α transcription requires estrogen receptor activation function-2 and is enhanced by coactivators. Proc Natl Acad Sci USA. (1999) 96:26. 10.1073/pnas.96.26.15161 PMC24790 10611355

[51] Chen CW Jian CY Lin PH Chen CC Lieu FK Soong C Role of testosterone in regulating induction of TNF-α in rat spleen via ERK signaling pathway. Steroids. (2016) 111:148–54. 10.1016/j.steroids.2016.03.007 26996389

[52] Stumper A Moriarity DP Coe CL Ellman LM Abramson LY Alloy LB. Pubertal Status and age are differentially associated with inflammatory biomarkers in female and male adolescents. J Youth Adolesc. (2020) 49:7. 10.1007/s10964-019-01101-3 PMC7015802 31410721

[53] Wiegering V Eyrich M Wunder C Günther H Schlegel PG Winkler B. Age-related changes in intracellular cytokine expression in healthy children. Eur Cytokine Netw. (2009) 20:2. 10.1684/ecn.2009.0149 19541593

[54] Fox HS Bond BL Parslow TG. Estrogen regulates the IFN-gamma promoter. The Journal of Immunology. (1991) 146:12. 1904081

[55] Zhang JM An J. Cytokines, inflammation, and pain. Int Anesthesiol Clin. (2007) 45:2. 10.1097/AIA.0b013e318034194e PMC2785020 17426506

[56] Kanda N Tamaki K. Estrogen enhances immunoglobulin production by human PBMCs. J Allergy Clin Immunol. (1999) 103:2. 10.1016/S0091-6749(99)70503-8 9949320

[57] Liva SM Voskuhl RR. Testosterone acts directly on CD4+ T lymphocytes to increase IL-10 production. J Immunol. (2001) 167:4. 10.4049/jimmunol.167.4.2060 11489988

[58] von Rango U Alfer J Kertschanska S Kemp B Müller-Newen G Heinrich PC Interleukin-11 expression: its significance in eutopic and ectopic human implantation. Mol Hum Reprod. (2004) 10(11):783–92. 10.1093/molehr/gah107 15465850

[59] Karpuzoglu-Sahin E Zhi-Jun Y Lengi A Sriranganathan N Ansar Ahmed S. Effects of long-term estrogen treatment on IFN*γ*, ILl-2 and IL-4 gene expression and protein synthesis in spleen and thymus of normal C57BL/6 mice. In: Cytokine. (2001) 14(4):208–17. 10.1006/cyto.2001.0876 11448120

[60] Blais Y Gingras S Haagensen DE Labrie F Simard J. Interleukin-4 and interleukin-13 inhibit estrogen-induced breast cancer cell proliferation and stimulate GCDFP-15 expression in human breast cancer cells. In: Molecular and Cellular Endocrinology. (1996) 121(1):11–8. 10.1016/0303-7207(96)03843-9 8865161

[61] Mantovani R Mantovani L Dias V. Thyroid autoimmunity in children and adolescents with type 1 diabetes Mellitus: prevalence and risk factors. J Pediatr Endocrinol and Metabolism. (2007) 20(6):669–76. 10.1515/JPEM.2007.20.6.669 17663291

[62] Singh RP Hahn BH Bischoff DS. Interferon genes are influenced by 17*β*-estradiol in SLE. Front Immunol. (2021) 12:725325. 10.3389/fimmu.2021.725325 34733276 PMC8558410

[63] Kassi E Moutsatsou P. Estrogen receptor signaling and its relationship to cytokines in systemic lupus erythematosus. J Biomed Biotechnol. (2010) 2010:317452. 10.1155/2010/317452 20617147 PMC2896666

[64] Zandman-Goddard G Peeva E Shoenfeld Y. Gender and autoimmunity. Autoimmun Rev. (2007) 6:6. 10.1016/j.autrev.2006.10.001 17537382

[65] Ortona E Pierdominici M Maselli A Veroni C Aloisi F Shoenfeld Y. Sex-based differences in autoimmune diseases. Ann Ist Super Sanita. (2016) 52:2. 10.4415/ANN_16_02_12 27364395

[66] Cutolo M Sulli A Capellino S Villaggio B Montagna P Seriolo B Sex hormones influence on the immune system: basic and clinical aspects in autoimmunity. Lupus. (2004) 13:9. 10.1191/0961203304lu1094oa 15485092

[67] Beato M Sánchez-Pacheco A. Interaction of steroid hormone receptors with the transcription initiation complex. Endocr Rev. (1996) 17:6. 10.1210/edrv-17-6-587 8969970

[68] Green NM Marshak-Rothstein A. Toll-like receptor driven B cell activation in the induction of systemic autoimmunity. Semin Immunol. (2011) 23:2. 10.1016/j.smim.2011.01.016 21306913 PMC3070769

[69] Benten WP Stephan C Wunderlich F. B cells express intracellular but not surface receptors for testosterone and estradiol. Steroids. (2002) 67:7. 10.1016/s0039-128x(02)00013-2 11996938

[70] Lü FX Abel K Ma Z Rourke T Lu D Torten J The strength of B cell immunity in female rhesus macaques is controlled by CD8+ T cells under the influence of ovarian steroid hormones. Clin Exp Immunol. (2002) 128:1. 10.1046/j.1365-2249.2002.01780.x 11982585 PMC1906365

[71] Tan IJ Peeva E Zandman-Goddard G. Hormonal modulation of the immune system - A spotlight on the role of progestogens. Autoimmun Rev. (2015) 14:6. 10.1016/j.autrev.2015.02.004 25697984

[72] Hughes GC. Progesterone and autoimmune disease. Autoimmun Rev. (2012) 6:7. 10.1016/j.autrev.2011.12.003 PMC3431799 22193289

[73] Hughes GC Choubey D. Modulation of autoimmune rheumatic diseases by oestrogen and progesterone. Nat Rev Rheumatol. (2014) 10:12. 10.1038/nrrheum.2014.144 25155581

[74] Burger HG. Androgen production in women. Fertil Steril. (2002) 77(Suppl 4):S3–5. 10.1016/s0015-0282(02)02985-0 12007895

[75] Prall SP Muehlenbein MP. DHEA Modulates immune function: a review of evidence. Vitam Horm. (2018) 108:125–44. 10.1016/bs.vh.2018.01.023 30029724

[76] Wilder RL. Adrenal and gonadal steroid hormone deficiency in the pathogenesis of rheumatoid arthritis. J Rheumatol Suppl. (1996) 44:10–2. 8833045

[77] Firestein GS McInnes IB. Immunopathogenesis of rheumatoid arthritis. Immunity. (2017) 46(2):183–96. 10.1016/j.immuni.2017.02.006 28228278 PMC5385708

[78] Cutolo M Seriolo B Villaggio B Pizzorni C Craviotto C Sulli A. Androgens and estrogens modulate the immune and inflammatory responses in rheumatoid arthritis. Ann N Y Acad Sci. (2002) 966:131–42. 10.1111/j.1749-6632.2002.tb04210.x 12114267

[79] Mackworth-Young CG Parke AL Morley KD Fotherby K Hughes GR. Sex hormones in male patients with systemic lupus erythematosus: a comparison with other disease groups. Eur J Rheumatol Inflamm. (1983) 6:3. 6439564

[80] Walecki M Eisel F Klug J Baal N Paradowska-Dogan A Wahle E Androgen receptor modulates Foxp3 expression in CD4 + CD25 + Foxp3 + regulatory T-cells. Mol Biol Cell. (2015) 26(15):2845–57. 10.1091/mbc.E14-08-1323 26063731 PMC4571343

[81] Yang Z Shen Y Oishi H Matteson EL Tian L Goronzy JJ Restoring oxidant signaling suppresses proarthritogenic T cell effector functions in rheumatoid arthritis. Sci Transl Med. (2016) 8(331):331ra38. 10.1126/scitranslmed.aad7151 27009267 PMC5074090

[82] Gamat M McNeel DG. Androgen deprivation and immunotherapy for the treatment of prostate cancer. Endocr Relat Cancer. (2017) 24(12):T297–310. 10.1530/ERC-17-0145 28814451 PMC5669826

[83] Rey RA. The role of androgen signaling in male sexual development at puberty. Endocrinology. (2021) 162(2):bqaa215. 10.1210/endocr/bqaa215 33211805

[84] Santi M Graf S Zeino M Cools M Van De Vijver K Trippel M Approach to the virilizing girl at puberty. J Clin Endocrinol Metab. (2021) 106(5):1530–9. 10.1210/clinem/dgaa948 33367768 PMC8063244

[85] Odani T Chiorini JA. Targeting primary Sjögren's Syndrome. Mod Rheumatol. (2019) 29:1. 10.1080/14397595.2018.1546268 30424703

[86] Manoussakis MN Tsinti M Kapsogeorgou EK Moutsopoulos HM. The salivary gland epithelial cells of patients with primary Sjögren's Syndrome manifest significantly reduced responsiveness to 17*β*-estradiol. J Autoimmun. (2012) 39:1–2. 10.1016/j.jaut.2012.01.005 22309821

[87] Ishimaru N Arakaki R Watanabe M Kobayashi M Miyazaki K Hayashi Y. Development of autoimmune exocrinopathy resembling Sjögren's Syndrome in estrogen-deficient mice of healthy background. Am J Pathol. (2003) 163:4. 10.1016/S0002-9440(10)63505-5 PMC1868285 14507655

[88] McCoy SS Sampene E Baer AN. Association of Sjögren's Syndrome with reduced lifetime sex hormone exposure: a case-control study. Arthritis Care Res. (2020) 72:9. 10.1002/acr.24014 PMC6928446 31233285

[89] Mavragani CP Fragoulis GE Moutsopoulos HM. Endocrine alterations in primary Sjogren's Syndrome: an overview. J Autoimmun. (2012) 39:4. 10.1016/j.jaut.2012.05.011 22695186

[90] Riese RJ Wolf PR Brömme D Natkin LR Villadangos JA Ploegh HL Essential role for cathepsin S in MHC class II-associated invariant chain processing and peptide loading. Immunity. (1996) 4:4. 10.1016/s1074-7613(00)80249-6 8612130

[91] Nakajima T Nakamura H Owen CA Yoshida S Tsuduki K Chubachi S Plasma cathepsin S and cathepsin S/cystatin C ratios are potential biomarkers for COPD. Dis Markers. (2016) 2016:4093870. 10.1155/2016/4093870 27994288 PMC5138488

[92] Wilkinson RD Williams R Scott CJ Burden RE. Cathepsin S: therapeutic, diagnostic, and prognostic potential. Biol Chem. (2015) 396:8. 10.1515/hsz-2015-0114 25872877

[93] Morthen MK Tellefsen S Richards SM Lieberman SM Rahimi Darabad R Kam WR Testosterone influence on gene expression in lacrimal glands of mouse models of sjögren syndrome. Invest Ophthalmol Vis Sci. (2019) 60:6. 10.1167/iovs.19-26815 PMC6528840 31108549

[94] Lee HJ Li CW Hammerstad SS Stefan M Tomer Y. Immunogenetics of autoimmune thyroid diseases: a comprehensive review. J Autoimmun. (2015) 64:82–90. 10.1016/j.jaut.2015.07.009 26235382 PMC4628844

[95] Kyritsi EM Kanaka-Gantenbein C. Autoimmune thyroid disease in specific genetic syndromes in childhood and adolescence. Front Endocrinol (Lausanne). (2020) 11:543. 10.3389/fendo.2020.00543 32973676 PMC7466763

[96] Cheng CW Fang WF Tang KT Lin JD. Possible interplay between estrogen and the BAFF may modify thyroid activity in Graves’ disease. Sci Rep. (2021) 11(1):21350. 10.1038/s41598-021-00903-5 34725405 PMC8560878

[97] Antonelli A Ferrari SM Ragusa F Elia G Paparo SR Ruffilli I Graves’ disease: epidemiology, genetic and environmental risk factors and viruses. Best Pract Res Clin Endocrinol Metab. (2020) 34(1):101387. 10.1016/j.beem.2020.101387 32107168

[98] Zhang Y Tian J Xiao F Zheng L Zhu X Wu L B cell-activating factor and its targeted therapy in autoimmune diseases. Cytokine Growth Factor Rev. (2022) 64:57–70. 10.1016/j.cytogfr.2021.11.004 34916133

[99] Thompson JS Schneider P Kalled SL Wang L Lefevre EA Cachero TG BAFF Binds to the tumor necrosis factor receptor-like molecule B cell maturation antigen and is important for maintaining the peripheral B cell population. J Exp Med. (2000) 192:129–35. 10.1084/jem.192.1.129 10880534 PMC1887706

[100] Biondi B Kahaly GJ Robertson RP. Thyroid dysfunction and diabetes Mellitus: two closely associated disorders. Endocr Rev. (2019) 40(3):789–824. 10.1210/er.2018-00163 30649221 PMC6507635

[101] Mogri M Dhindsa S Quattrin T Ghanim H Dandona P. Testosterone concentrations in young pubertal and post-pubertal obese males. Clin Endocrinol (Oxf). (2013) 78:4. 10.1111/cen.12018 PMC3524388 22970699

[102] Fournier JP Yin H Yu OHY Azoulay L. Metformin and low levels of thyroid-stimulating hormone in patients with type 2 diabetes mellitus. Can Med Assoc J. (2014) 186(15):1138–45. 10.1503/cmaj.140688 25246411 PMC4203599

[103] Faulkner JL Belin de Chantemèle EJ. Sex hormones, aging and cardiometabolic syndrome. Biol Sex Differ. (2019) 10:1. 10.1186/s13293-019-0246-6 31262349 PMC6604485

[104] Silverman ED Isacovics B Petsche D Laxer RM. Synovial fluid cells in juvenile arthritis: evidence of selective T cell migration to inflamed tissue. Clin Exp Immunol. (1993) 91(1):90–5. 10.1111/j.1365-2249.1993.tb03360.x 8093436 PMC1554635

[105] Kawai T Autieri MV Scalia R. Adipose tissue inflammation and metabolic dysfunction in obesity. Am J Physiol Cell Physiol. (2021) 320(3):C375–91. 10.1152/ajpcell.00379.2020 33356944 PMC8294624

[106] de Candia P Prattichizzo F Garavelli S De Rosa V Galgani M Di Rella F Type 2 diabetes: how much of an autoimmune disease? Front Endocrinol. (2019) 10:451. 10.3389/fendo.2019.00451 PMC6620611 31333589

[107] Snir O Widhe M von Spee C Lindberg J Padyukov L Lundberg K Multiple antibody reactivities to citrullinated antigens in sera from patients with rheumatoid arthritis: association with HLA-DRB1 alleles. Ann Rheum Dis. (2009) 68(5):736–43. 10.1136/ard.2008.091355 18635594

[108] Khalkhali-Ellis Z Moore TL Hendrix MJ. Could hormones make a difference in the treatment of juvenile rheumatoid arthritis? BioDrugs. (2000) 13(2):77–86. 10.2165/00063030-200013020-00001 18034514

[109] Poulsen TBG Damgaard D Jorgensen MM Senolt L Blackburn JM Nielsen LH. Identification of novel native autoantigens in rheumatoid arthritis. Biomedicines. (2020) 8(6 ):141. 10.3390/biomedicines8060141 32486012 PMC7345460

[110] Thompson SD Murray KJ Grom AA Passo MH Choi E Glass DN. Comparative sequence analysis of the human T cell receptor beta chain in juvenile rheumatoid arthritis and juvenile spondylarthropathies: evidence for antigenic selection of T cells in the synovium. Arthritis Rheum. (1998) 41(3):482–97. 10.1002/1529-0131(199803)41:3<482::AID-ART15>3.0.CO;2-G 9506577

[111] Karamifar H Habibian N Amirhakimi G Karamizadeh Z Alipour A. Adiponectin is a good marker for metabolic state among type 1 diabetes Mellitus patients. Iran J Pediatr. (2013) 23:3. PMC3684474 23795252

[112] Maric C. Sex, diabetes and the kidney. Am J Physiol Renal Physiol. (2009) 296:4. 10.1152/ajprenal.90505.2008 PMC2670637 19144692

[113] Kelsey MM Bjornstad P McFann K Nadeau K. Testosterone concentration and insulin sensitivity in young men with type 1 and type 2 diabetes. Pediatr Diabetes. (2016) 17:3. 10.1111/pedi.12255 PMC4510044 25611822

[114] Moriarty-Kelsey M Harwood JE Travers SH Zeitler PS Nadeau KJ. Testosterone, obesity and insulin resistance in young males: evidence for an association between gonadal dysfunction and insulin resistance during puberty. J Pediatr Endocrinol Metab. (2010) 23:12. 10.1515/jpem.2010.202 PMC6112157 21714462

[115] Raziuddin S Bahabri S Al-Dalaan A Siraj AK Al-Sedairy S. A mixed Th1/Th2 cell cytokine response predominates in systemic onset juvenile rheumatoid arthritis: immunoregulatory IL-10 function. Clin Immunol Immunopathol. (1998) 86(2):192–8. 10.1006/clin.1997.4457 9473382

[116] Wedderburn LR Woo P. Type 1 and type 2 immune responses in children: their relevance in juvenile arthritis. Springer Semin Immunopathol. (1999) 21:361–74. 10.1007/BF00812262 10666778

[117] Cavalcanti A Santos R Mesquita Z Duarte AL Lucena-Silva N. Cytokine profile in childhood-onset systemic lupus erythematosus: a cross-sectional and longitudinal study. Braz J Med Biol Res. (2017) 50(4):e5738. 10.1590/1414-431X20175738 28380214 PMC5423750

[118] Zerwas S Larsen JT Petersen L Thornton LM Quaranta M Koch SV Eating disorders, autoimmune, and autoinflammatory disease. Pediatrics. (2017) 140:6. 10.1542/peds.2016-2089 PMC5703777 29122972

[119] Raevuori A Haukka J Vaarala O Suvisaari JM Gissler M Grainger M The increased risk for autoimmune diseases in patients with eating disorders. PLoS One. (2014) 9:8. 10.1371/journal.pone.0104845 PMC4141740 25147950

